# Patient perceived service quality profiles of primary health care associated with usual source of care: a latent profile analysis

**DOI:** 10.3389/fmed.2025.1592803

**Published:** 2025-06-26

**Authors:** Li Zhang, Baokai Wang, Qi Xu

**Affiliations:** ^1^School of Health Management, Binzhou Medical University, Yantai, Shandong, China; ^2^Yantai Yuhuangding Hospital, Yantai, Shandong, China; ^3^School of Public Health, Binzhou Medical University, Yantai, Shandong, China

**Keywords:** perceived service quality, usual source of care, latent profile analysis, primary health care, cross-sectional survey

## Abstract

**Background:**

Under the context that the Chinese government has taken many incentives to promote the development of primary health care (PHC), the extent to which patients choose PHC as their usual source of care (USC) and how patient perceived service quality affects this choice remains unclear. This study aimed to explore potential profiles of patient perceived service quality of PHC, analyse sociodemographic and health-related characteristics among perceived service quality subgroups, and investigate the association between patient perceived service quality and usual source of PHC.

**Methods:**

A cross-sectional survey was conducted in Shandong Province, China, from July 2023 to August 2023. 3025 patients were selected using a multistage stratified sampling method. Latent profile analysis was used to explore the profiles of patient perceived service quality of PHC. Multiple logistic regression analysis was adopted to identify the predictors of different profiles, and the binary logistic regression was used to examine the association between perceived service quality profiles and usual source of PHC.

**Results:**

The model fitting indices of patient perceived service quality supported three profiles which were as follows: low perceived service quality group at 20.23%, moderate perceived service quality group at 56.50%, and high perceived service quality group at 23.27%. Age, educational level, personal monthly income, and self-rated health were predictors of different profiles. Furthermore, patients reporting high perceived service quality of PHC were more likely to regard PHC as the USC compared with those with low perceived service quality (OR = 2.748, 95% confidence interval = 2.112–3.575).

**Conclusion:**

Strengthening patient perceived service quality can encourage them to regard PHC facilities as the USC. PHC facilities should provide customized and tailored health services that focus on individuals’ specific needs and preferences to improve patient perceived service quality.

## Introduction

Usual source of care (USC) refers to the provider or place that a person or family usually consults when sick or in need of medical or health advice, implying an ongoing relationship with a health care provider or facility (1, 2). Robust evidence demonstrates that individuals with a USC are more likely to experience improved health care accessibility, increased use of preventive services, reduced hospitalizations and emergency department visits, fewer unmet health needs, decreased health care disparities, lower medical expenditure, improved health behaviors, better health status, and increased trust and satisfaction with health care providers compared with those without a USC (3–8). On the contrary, the lack of USC is related to negative outcomes, such as reduced access to health care, increased risk of non-urgent emergency department visits and hospitalizations, and decreased patient experience with health care (4, 9).

China has a three-tier health care system consisting of primary health care (PHC) facilities, secondary hospitals, and tertiary hospitals. People in China have the freedom to choose any health care institutions for medical and health services at any time without being referred by PHC doctors (9, 10). Therefore, patients can select any type of health care provider or facility as their USC. PHC, considered the foundation and guarantee of an efficient health care system, has been considered worldwide to contribute to better population health, greater efficiency and equity in health care, and lower costs (9, 11). However, historical underinvestment, workforce shortages and skill gaps in PHC, systemic coordination and integration challenges, and other factors in China have led to a significant trend where most patients bypass PHC institutions and directly seek services at higher level hospitals due to perceived superior medical technology and higher service quality, regardless of the severity of their symptoms (10, 12). Consequently, higher level hospitals become overcrowded, while PHC facilities remain underdeveloped and struggle to fulfill their functions (12, 13). When secondary or tertiary hospitals are consistently the usual choice for patients to seek medical and health services, even for minor ailments, problems such as prolonged patients waiting times, reduced patient involvement in health care, inappropriate health service utilization, and increased medical expenditure will arise (4, 12–14).

To address these problems, China has significantly increased its efforts to strengthen PHC, particularly since the 2009 health care reform. Strengthening PHC has been among the top priorities guiding health care reforms (13, 14). These efforts involve a comprehensive set of measures, including substantial investments on capital, equipment, and human resources, as well as policies aimed at strengthening PHC and attracting a wide range of patients (10, 15). Under China’s health care reform, PHC facilities and PHC physicians have been considered as the preferred medical or health service providers, making health care more accessible to patients. Therefore, under this context, it is crucial to draw public attention to the question of how many patients choose PHC as their USC and to clarify the factors influencing the lack of usual source of PHC in China to promote the development of PHC.

Health service quality has become an important strategic focus for health care organizations worldwide, particularly with the growing competition in the health care industry (16, 17). Furthermore, quality assessment based on patients’ views, experiences, and perceptions has been emphasized in the patient-centered service model (18, 19). Thus, evaluating patients’ perceptions of health services has become more important than ever before (20). Patient perceived service quality has been descripted as patient’s perception level of impression or experience regarding the provided health services. It commonly refers to the discrepancies and mismatches between individuals’ prior expectations for a service offering and their actual perceptions about the quality of service received (21–23). The value of patient perceived service quality has been increasingly recognized in health care by previous studies, as it promotes patients’ perceived value, satisfaction, and their behavioral intentions, including recommendations and return visits (3, 21, 24). Moreover, empirical research has shown that patient’s perception of health service quality helps health care managers allocate resources more effectively and ensures high patient utilization of services (20).

In the context of government incentives aimed at fostering the development of PHC, while numerous studies have examined perceptions of primary health service quality, the effect of patient perceived service quality on their usual source of PHC remains underexplored. In addition, most previous studies have evaluated patient perceived service quality based on the total or mean scores, overlooking individual factors and leading to significant heterogeneity across categories. Latent profile analysis (LPA) is an individual-centered statistical method that can address this heterogeneity by categorizing samples based on specific item characteristics. Therefore, in the present study, patient perceived service quality subgroups were first classified using LPA. Subsequently, we examined the sociodemographic and health-related characteristics associated with these latent profiles. Finally, we investigated the relationship between patient perceived service quality subgroups and usual source of PHC. Our findings could prompt health policymakers to develop mechanisms to enhance service quality and implement interventions to promote PHC utilization.

## Materials and methods

### Study design and participants

A cross-sectional study was carried out between July and August 2023 in Shandong Province, which is located in eastern China and is one of China’s typical economically developed and densely populated regions. The multistage stratified random sampling method was adopted to select PHC facilities in 16 prefectures of Shandong Province. In the first stage, two counties or districts were randomly selected in each prefecture. In the second stage, we stratified urban and rural areas within each district or county, such that one street and one township were randomly selected in each district or county. In the third stage, one community health service center and one township hospital were randomly selected in each street or township. In total, 64 PHC facilities were chosen for investigation in this study. A convenience sampling method was adopted to choose participants to explore the services provided by the selected institution. All surveys were conducted face-to-face interviews by postgraduate and undergraduate students trained by researchers from the School of Health Management at Binzhou Medical University.

Eligible participants were patients aged 18 years and above without cognition impairment, and had visited the study sites before surveyed. Those patients were invited to participate in the survey after a fully explanation of the study purpose and were informed that they would receive a small gift upon completion of the questionnaire. Exclusion criteria were as follows: (1) patients with communication barriers, (2) those had difficulty understanding the questionnaire content, (3) those who had never visited the surveyed institution. According to the standard sample size formula for the cross-sectional survey and previous related research indicating that 35.7% of patients regarded the PHC facility as their USC (9), the minimum sample size for our study was calculated at 392, with a 95% confidence interval, an absolute error of 5%, and a refusal rate of 10%. We collected 3149 questionnaires, of which 124 were excluded due to logical errors or participants dropping out during the study. Therefore, 3025 responses were analyzed, with a valid questionnaire rate of 96.06%. This study was approved by the Ethics Committee of Binzhou Medical University (2021-337).

### Measurement

#### Perceived service quality

The adapted and validated Chinese version of the SERVQUAL instrument was used to evaluate patient’s perception of primary health service quality (25). This scale consists of 17 items and six dimensions: tangibility, reliability, responsiveness, assurance, empathy, and cost acceptability. Participants answered each item on a five-point Likert scale (1 = poor, 5 = excellent). The mean score of all items represented the overall patient perceived service quality. Higher scores indicated greater levels of patient’s perception with primary health service quality.

#### Usual source of PHC

Based on the classification system provided by the Primary Care Assessment Tool (PCAT), and the opinions of previous researchers (2, 4, 9), the usual source of PHC was assessed by participant’s response to the following questions: (1) Is there a PHC facility to which you usually go, when you are sick or need advice about your health? (2) Is there a PHC facility that knows you best? (3) Is there a PHC facility that is most responsible for your health care? Individuals who responded ‘yes’ to any one of these questions were identified as having a usual source of PHC; while those who responded ‘no’ to all three questions were defined as not having a usual source of PHC.

#### Other variables

The questionnaire also collected questions on participants’ sociodemographic data (e.g., age, gender, marital status, residency, ethnicity, occupation status, level of education, personal monthly income, medical insurance) and health-related characteristics (e.g., self-rated health, chronic disease, anxiety, depression). These variables were selected based on their potential associations with outcome variables and previous related studies (3, 4, 9, 26).

### Statistical analysis

Data in our study were analyzed using the IBM SPSS version 24.0 and Mplus version 8.3. Firstly, frequency and percentage, mean, and standard deviation were adopted to calculate descriptive statistics for all variables. Secondly, for the LPA of patients’ perceived service quality of PHC, the 17 items of perceived service quality were selected as observed variables. The initial model assumed all participants to be in one category, and the latent class was successively added until the model fit reached optimal requirements. The main model fitting indices including the Akaike information criterion (AIC), Bayesian information criterion (BIC), adjusted Bayesian information criterion (aBIC), Entropy index, Lo-Mendell-Rubin likelihood ratio test (LMR), and Bootstrapped likelihood ratio test (BLRT) were used to determine the best model. Lower values for the AIC, BIC, and aBIC indicate a better-fitting model. Entropy varies between 0 and 1, with larger values indicating better classification accuracy. Significant *P* values for LMR and BLRT indicate that the model with k profiles is more appropriate than the model with k-1 profiles. In addition to the above indicators, practical significance, interpretability, and the sample size of each profile (proportion of each category ≥ 5%) were also considered (27–29). Thirdly, the multiple logistic regression analysis was adopted to explore the predictors of perceived service quality, with profile as the dependent variable and the sociodemographic and health-related factors as independent variables. Finally, the binary logistic regression was used to examine the association between perceived service quality profiles and usual source of PHC, after controlling for sociodemographic and health-related variables that affect usual source of PHC.

## Results

### Sample characteristics

The sociodemographic and health-related characteristics of the participants are shown in Table 1. The average age of patients was 43.85 ± 17.87 years, with 41.85% male and 58.15% female participants. Among them, 213 (7.04%) were divorced or widowed, and 856 (28.30%) were unmarried. Moreover, 2188 (72.33%) were rural patients and 837 (27.67%) were urban patients. Most of the participants were Han (99.37%). Of them, 1153 (38.12%) were unemployed. More than half of the participants (58.45%) had a senior high school education or below. Patients who reported a personal monthly income of 3,000 RMB or less accounted for 69.69% (*n* = 2,108). Only 63 (2.08%) participants were not covered by medical insurance. A total of 2054 (67.90%) patients reported good health status, while 980 (32.40%) patients reported having at least one chronic disease. A small number of participants reported experiencing anxiety (7.34%) or depression (7.24%).

**TABLE 1:** Sociodemographic and health-related characteristics of the study participants (*n* = 3,025).

Characteristic		N	%
**Gender**	Male	1,266	41.85
	Female	1,759	58.15
**Age**	(Mean ± SD)	43.85 ± 17.87	
	18–39 years	1227	40.56
	40–59 years	1,174	38.81
	≥ 60 years	624	20.63
**Marital status**	Married	1,956	64.66
	Divorced or widowed	213	7.04
	Not married	856	28.30
**Residency**	Rural	2,188	72.33
	Urban	837	27.67
**Ethnicity**	Han	3,006	99.37
	Others	19	0.63
**Occupation status**	Have a job	1,872	61.88
	Have no job	1,153	38.12
**Educational level**	Bachelor’s level or above	850	28.10
	College	407	13.45
	Senior high school or below	1,768	58.45
**Personal monthly income**	≤ 3,000 RMB	2,108	69.69
	> 3,000 RMB	917	30.31
**Medical insurance**	No	63	2.08
	Basic medical insurance	2,775	91.74
	Other medical insurance	187	6.18
**Self-rated health**	Poor	230	7.60
	Moderate	741	24.50
	Good	2,054	67.90
**Chronic disease**	No	2,045	67.60
	Yes	980	32.40
**Anxiety**	No	2,803	92.66
	Yes	222	7.34
**Depression**	No	2,806	92.76
	Yes	219	7.24

SD, standard deviation.

### Latent profile analysis of perceived service quality

To identify classes with minimal variation within subgroups and as much variation between subgroups as possible, we performed the LPA with 17 items of perceived service quality entered into the model individually. Our exploratory LPA was run with one to five solutions to determine the best model fit. Information on each model fit indices are presented in Table 2. As the number of categories increased, the values of AIC, BIC, and aBIC continuously decreased. Both the LMR and the BLRT for models two to five were statistically significant (*P* < 0.001). Despite the entropy value suggesting that profile 4 (0.984) or profile 5 (0.981) may fit better than profile 3 (0.980), however, the smallest subgroup size was less than 5% of the total sample for the four-profile model or the five-profile model. While profile 3 had a higher entropy value compared to the profile 2 (0.974), we ultimately selected the three-profile model as the best-fitting result after considering the model fit indices, the interpretability, and practical significance. Thus, patient perceived service quality was classified into three latent profiles.

**TABLE 2:** Model fit indices of the latent profile analysis of perceived service quality (*n* = 3,025).

Model	AIC	BIC	aBIC	Entropy	LMR (P)	BLRT (P)	Conditional probability
1	12,4020.806	12,4225.305	12,4117.273				
2	98,239.12/5	98,551.888	98,386.664	0.974	<.001	<0.001	0.261/0.739
3	78234.350	78,655.377	78,432.959	0.980	<0.001	<0.001	0.202/0.565/0.233
4	69,172.721	69,702.011	69,422.401	0.984	0.0007	<0.001	0.017/0.545/0.209/ 0.229
5	66,535.214	67,172.768	66,835.965	0.981	<0.001	<0.001	0.017/0.201/0.498/ 0.105/0.179

AIC, akaike information criterion; BIC, bayesian information criterion; aBIC, adjusted BIC; LMR, Lo-Mendell-Rubin likelihood ratio test; BLRT, Bootsrapped likelihood ratio test.

**FIGURE 1:**
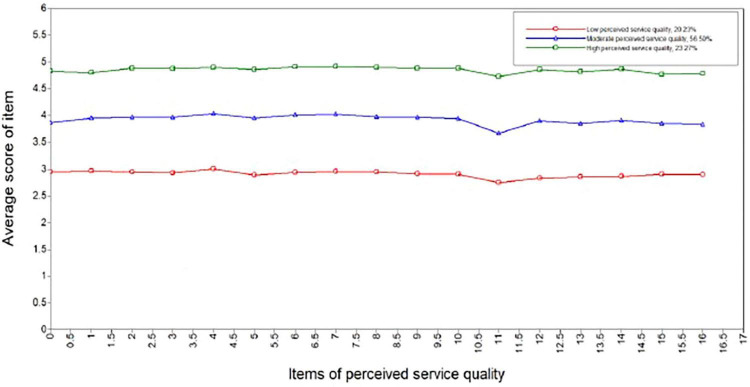
The latent profiles of patient perceived service quality with primary health care (*n* = 3025).

Figure 1 illustrates the distribution of the average scores of items of perceived service quality classified into three categories. Profile 1 (*n* = 612, 20.23% of the sample) consisted of individuals with extremely low average scores of each item, labeled as ‘low perceived service quality’. Profile 2 (*n* = 1709), accounted for 56.50% of all samples, was characterized by a moderate average score of the items, hence named ‘moderate perceived service quality’. Profile 3, encompassing 23.27% of the sample (*n* = 704), was broadly characterized by high average scores across all items, and was labeled as ‘high perceived service quality’.

### Characteristics of the latent classes of perceived service quality

The results of the multinomial logistic regression analysis to explore the factors influencing each subgroup of perceived service quality are presented in Table 3. Gender, age, marital status, residency, ethnicity, occupation status, educational level, personal monthly income, medical insurance, chronic disease status, self-rated health, anxiety, and depression were included as the independent variables, with the latent profiles of perceived service quality serving as the dependent variables, using the high perceived service quality class as a reference. The results indicated that patients aged 18–39 (odds ratio [OR] = 1.991, 95% confidence interval [CI] = 1.266–3.131) or 40–59 (OR = 2.046, 95% CI = 1.409–2.972), those reporting a personal monthly income of 3,000 RMB or less (OR = 1.426, 95% CI = 1.102–1.845), and those experiencing depression (OR = 2.001, 95% CI = 1.138–3.519) were more likely to be in the low perceived service quality group. In addition, individuals with a college (OR = 0.631, 95% CI = 0.436–0.913) or bachelor’s degree and above (OR = 0.655, 95% CI = 0.464–0.923), as well as those with good self-perceived health status (OR = 0.431, 95% CI = 0.248–0.749), were less likely to have low perceived service quality compared with those with a high school education or below, and those with poor self-perceived health status.

**TABLE 3:** Sociodemographic and health-related characteristics associated with the three latent phenotypes of patient perceived service quality.

Characteristics	Low perceived service quality			Moderate perceived service quality		
	*B*	*OR*	95%CI	*B*	OR	95%CI
**Gender (Reference: Female)**						
Male	0.082	1.085	(0.865,1.361)	–0.032	0.969	(0.807,1.163)
**Age (Reference: ≥ 60 years)**						
18–39 years	0.688	1.991	(1.266,3.131)	–0.186	0.831	(0.584,1.181)
40–59 years	0.716	2.046	(1.409,2.972)	0.137	1.146	(0.861,1.526)
**Marital status (Reference: Divorced or widowed)**						
Married	–0.104	0.901	(0.539,1.506)	–0.207	0.813	(0.541,1.223)
Not married	0.073	1.076	(0.583,1.985)	0.342	1.408	(0.862,2.300)
**Residency (Reference: Rural)**						
Urban	–0.072	0.931	(0.718,1.205)	–0.169	0.844	(0.686,1.040)
**Ethnicity (Reference: Others)**						
Han	–0.130	0.878	(0.259,2.980)	0.569	1.767	(0.585,5.333)
**Occupation status (Reference: Have a job)**						
Have no job	0.171	1.186	(0.881,1.598)	–0.125	0.882	(0.696,1.119)
**Educational level (Reference: High school and below)**						
Bachelor’s level and above	–0.424	0.655	(0.464,0.923)	–0.319	0.727	(0.552,0.959)
College	–0.460	0.631	(0.436,0.913)	–0.367	0.693	(0.517,0.929)
**Personal monthly income (Reference: > 3,000 RMB)**						
≤ 3,000 RMB	0.355	1.426	(1.102,1.845)	0.032	1.032	(0.844,1.262)
**Medical insurance (Reference: Other medical insurance)**						
No	0.143	1.154	(0.559,2.385)	–0.846	0.429	(0.203,0.905)
Basic medical insurance	–0.021	0.979	(0.637,1.504)	0.344	1.410	(0.981,2.026)
**Chronic disease (Reference: Yes)**						
No	0.147	1.158	(0.835,1.606)	–0.064	0.938	(0.721,1.220)
**Self-rated health (Reference: Poor)**						
Good	–0.842	0.431	(0.248,0.749)	–0.811	0.444	(0.283,0.697)
Moderate	0.219	1.245	(0.719,2.156)	–0.287	0.750	(0.475,1.185)
**Anxiety (Reference: No)**						
Yes	0.173	1.189	(0.681,2.075)	–0.061	0.941	(0.569,1.555)
**Depression (Reference: No)**						
Yes	0.694	2.001	(1.138,3.519)	0.010	1.010	(0.599,1.703)

OR, odds ratio; CI, confidence interval; Ref, high perceived service quality.

### Association between perceived service quality and usual source of PHC

Table 4 shows that 30.02% of the patients regarded PHC facility as their usual source of care. We primarily used the Chi-square test to investigate the relationship between perceived service quality profiles and usual source of PHC. The results revealed that patients reporting high perceived service quality of PHC (35.80%) were more likely to have a usual source of PHC compared with those reporting low (18.14%) or moderate (31.89%) perceived service quality of PHC (*P* < 0.001).

**TABLE 4:** Association between the latent profile membership and usual source of primary health care.

Subgroups of perceived service quality	Primary health care as USC	Non-Primary health care as USC	χ^2^	*P*
Low perceived service quality	111 (18.14)	501 (81.86)	55.160	< 0.001
Moderate perceived service quality	545 (31.89)	1164 (68.11)		
High perceived service quality	252 (35.80)	452 (64.20)		
Total	908 (30.02)	2117 (69.98)	-	-

Binary logistic regression analysis was performed further to test the association between perceived service quality profiles and usual source of PHC. First, simple logistic regression analysis was adopted to identify which sociodemographic and health-related characteristics affected usual source of PHC. Covariates including age, marital status, residency, ethnicity, educational level, personal monthly income, medical insurance, and chronic disease status yielded significant results and were included in the binary logistic regression model as the confounding factors.

Table 5 provides the results of the binary logistic regression for the association between perceived service quality profiles and usual source of PHC after controlling for related variables. Patients reporting high (OR = 2.748, 95% CI = 2.112–3.575) or moderate perceived service quality (OR = 2.060, 95% CI = 1.630–2.604) of PHC were more likely to regarding PHC as their USC.

**TABLE 5:** Logistic regression analysis for usual source of primary health care related variables.

Variables	*B*	*SE*	Waldχ^2^	*P*	*OR*	95% *CI*
**Age (Reference: ≥ 60 years)**						
18–39 years	–0.467	0.160	8.539	0.003	0.627	(0.458,0.857)
40–59 years	–0.225	0.118	3.629	0.057	0.799	(0.634,1.006)
**Marital status (Reference: Divorced or widowed)**						
Married	0.107	0.166	0.412	0.521	1.112	(0.804,1.540)
Not married	0.021	0.208	0.010	0.919	1.021	(0.679,1.536)
**Residency (Reference: Rural)**						
Urban	–0.546	0.104	27.418	<0.001	0.579	(0.472,0.711)
**Ethnicity (Reference: Others)**						
Han	2.039	1.035	3.880	0.049	7.686	(1.010,58.477)
**Educational level (Reference: High school and below)**						
Bachelor’s level and above	0.050	0.135	0.136	0.712	1.051	(0.806,1.370)
College	–0.011	0.145	0.005	0.941	0.989	(0.745,1.315)
**Personal monthly income (Reference: > 3000 RMB)**						
≤ 3000 RMB	0.319	0.097	10.725	0.001	1.376	(1.137,1.665)
**Medical insurance (Reference: Other medical insurance)**						
No	0.476	0.345	1.906	0.167	1.609	(0.819,3.163)
Basic medical insurance	0.324	0.190	2.898	0.089	1.383	(0.952,2.009)
**Chronic disease (Reference: Yes)**						
No	–0.183	0.103	3.112	0.078	0.833	(0.680,1.020)
**Perceived service quality (Reference: Low perceived**						
**service quality)**						
Moderate perceived service quality	0.723	0.120	36.582	< 0.001	2.060	(1.630,2.604)
High perceived service quality	1.011	0.134	56.660	< 0.001	2.748	(2.112,3.575)

SE, standard error; OR, Odds ratio; CI, Confidence interval.

## Discussion

This study explored patient perceived service quality profiles and their associations with usual source of PHC using a cross-sectional survey of 3025 patients in Shandong Province, eastern China. The results classified patient perceived service quality into three latent profiles: low perceived service quality group (profile 1), moderate perceived service quality group (profile 2), and high perceived service quality group (profile 3) by using LPA. Younger patients, those reporting a personal monthly income of 3,000 RMB or less, individuals with a high school education or below, and those perceiving poor health status were more likely to have low perceived service quality of PHC. Furthermore, patients reporting high or moderate perceived service quality of PHC were more likely to consider PHC as their USC compared with those with low perceived service quality. This study adopted a new measure to evaluate perceived service quality, and suggests that interventions focusing on the relationship between patient perceived service quality and usual source of PHC could increase appropriate PHC utilization.

The classification of patient perceived service quality into three profiles—low, moderate, and high—reveals heterogeneity in how patients perceive primary health service quality. Our findings indicated that only 23.27% of participants were classified in the high perceived service quality group, with higher overall mean scores on the scale items, which suggests a need for improvement in patient perceived service quality of PHC. Patient’s perception of service quality reflects the satisfaction with the needs and expectations of health services, which can inform the design of quality improvement programs to increase patients’ intentions to utilize primary health services (3, 19, 20). Therefore, supportive policies and guidelines are required to improve technical and functional quality, eventually enhancing patients’ perceptions of PHC quality. This could include providing more support resources, such as equipment and physicians through substantial public financial investment; strengthening general practitioners’ capacity through standardized, specialized, and systematic training; and promoting connectivity between large hospitals and PHC facilities.

Multiple logistic regression analysis showed that age, educational level, personal monthly income, and self-rated health were found to be predictors of patient perceived service quality in different profiles. Patients with higher educational levels tended to report higher perceived service quality, which was consistent with the results of previous study (20). One possible explanation for this finding is that lower education may lead to more illogical expectations, while patients with higher educational levels tend to have more positive perceptions of service quality (20). Our results also showed that individuals in good health were more likely to perceive high PHC quality, which was in line with the finding of a previous research indicating that patients with better health tended to have lower expectations and more positive perceptions (20). Furthermore, older participants and those with higher personal monthly income had more favorable evaluations. These findings highlight statistically significant differences between patients’ perceptions of quality and their sociodemographic and health-related characteristics, suggesting that health care providers should offer more customized and tailored health services that focus on individuals’ specific needs and preferences.

Regarding the usual source of PHC, 30.02% of participants regarded PHC as their USC, which was lower than the rates reported in other countries, such as the United States of America and Japan (11, 30). However, data from a previous empirical study in China reported that only 34.39% of patients regarded PHC facilities as their USC (4). Another study conducted in community health service centers showed that 35.7% of the patients were considered to have a usual source of community health service (9). The differences in proportions among Chinese studies may be related to the geographic and economic factors influencing the development of PHC facilities. The significantly lower proportions recorded in these studies indicate that only a minority of patients regard PHC facilities as their USC in China. Previous research has indicated that investigate patient’s choice of USC is important for guiding the ongoing development of PHC-based integrated health system building in China (4). Patients choosing PHC facilities as their USC are more likely to experience better care accessibility, have a higher awareness of health status, and feel more confident in their health management (4). Therefore, there is a considerable need to improve the prevalence of usual source of PHC, especially given the incentives the government has implemented to promote the development of PHC.

In the analysis of the relationship between patient perceived service quality profiles and usual source of PHC, this study found that patients with high perceived service quality were more likely to have a usual source of PHC compared with those reporting low perceived service quality. This suggests that a high perception of health service quality facilitates patients regarding PHC facilities as the USC. This can be explained by the fact that perceived service quality that reflects how well health service delivery meets individuals’ needs, preferences, and values (31). When patients perceive that the quality of service meets or exceeds their expectations, they are more likely to increase their return visits and service utilization, ultimately, regarding PHC institutions as their USC. This finding provides a novel insight that perceived service quality is positively associated with usual source of PHC, which provides health managers an implication that patient perceived service quality plays an active role in promoting the status of usual source of PHC.

### Limitations and future research

While our study contributes to understanding the association between patient perceived service quality and usual source of PHC, it has several limitations in this study. First, the results of this cross-sectional study were associative, and causal inferences cannot be drawn. Future research should consider a longitudinal design. Second, while Shandong Province offers a diverse and representative sample, our research data were limited to one province in China, which may limit the generalizability of the findings. Future studies should consider to expand the sample to other regions to confirm the findings. Third, while self-reported surveys are widely used to evaluate health service quality, there may be recall bias in our study. Finally, while the 2023 survey data may be affected by subsequent events, our cross-sectional study establishes baseline insights into the relationship between patient perceived service quality and usual source of PHC, future research could replicate the study with updated data to assess longitudinal changes.

## Conclusion

This study provides empirical evidence on the relationship between perceived service quality and USC. The findings are valuable for PHC managers seeking to improve PHC utilization, suggesting that perceived service quality plays a crucial role in encouraging patients to regard PHC facilities as the USC. Strengthening patients’ perceived service quality can enhance the proportion of usual source of PHC. Therefore, the government and health managers should consider the interaction between perceived service quality and usual source of PHC in the development of PHC, and continue its ongoing efforts to improve primary health service quality. This includes implementing supportive policies and improving technical and functional quality, as well as providing more customized and tailored health services that focus on individuals’ specific needs and preferences by PHC providers.

## Data Availability

The raw data supporting the conclusions of this article will be made available by the authors, without undue reservation.
